# Improvements With Tiotropium Maintenance Therapy in COPD Patients Without Dyspnea

**DOI:** 10.1002/mco2.71001

**Published:** 2026-09-16

**Authors:** Zihui Wang, Junfeng Lin, Guannan Cai, Fan Wu, Zhishan Deng, Qi Wan, Gaoying Tang, Cuiqiong Dai, Lifei Lu, Kunning Zhou, Xiaohui Wu, Suyin Huang, Fangyan Wu, Nanshan Zhong, Yumin Zhou, Pixin Ran

**Affiliations:** ^1^ State Key Laboratory of Respiratory Disease & National Clinical Research Center for Respiratory Disease & National Center For Respiratory Medicine & Guangzhou Institute of Respiratory Health The First Affiliated Hospital of Guangzhou Medical University Guangzhou China; ^2^ Guangzhou National Laboratory Guangzhou China

**Keywords:** chronic obstructive pulmonary disease, clinically important deterioration, exacerbations, lung function, non‐dyspneic, tiotropium

## Abstract

Current Global Initiative for Chronic Obstructive Lung Disease (GOLD) recommendations emphasize symptom‐driven management, which may limit the use of maintenance bronchodilator therapy in patients without dyspnea. In this exploratory post hoc analysis of the tiotropium in early‐stage chronic obstructive pulmonary disease (Tie‐COPD) trial, we assessed tiotropium in 305 patients with mild‐to‐moderate chronic obstructive pulmonary disease (COPD) and a modified Medical Research Council dyspnea score of 0 (157 tiotropium, 148 placebo). Over 2 years, tiotropium maintained a higher pre‐bronchodilator forced expiratory volume in 1 s (FEV_1_) than placebo (adjusted between‐group differences 143–183 mL; all *p* < 0.001). The lung function benefit was similar across subgroups defined by age, body mass index, symptom burden, blood eosinophil count, GOLD stage, and smoking (all *p* for interaction >0.05). The annual exacerbation rate was lower with tiotropium (adjusted incidence rate ratio 0.51, 95% confidence interval [CI] 0.32, 0.82; *p* = 0.005), as was the risk of a first clinically important deterioration (adjusted hazard ratio 0.55, 95% CI 0.43, 0.70; *p* < 0.001), whereas COPD assessment test and clinical COPD questionnaire scores did not differ. These hypothesis‐generating findings indicate that tiotropium may provide benefits in lung function and exacerbations in COPD without dyspnea, suggesting that a strictly symptom‐based treatment criterion warrants prospective reevaluation.

## Graphical abstract

In a post hoc analysis of the Tie‐COPD trial, including 305 patients with mild‐to‐moderate COPD and no dyspnea (mMRC 0), 2 years of tiotropium improved lung function, lowered the annual exacerbation rate, and delayed the first clinically important deterioration, indicating benefit beyond symptom relief in a group not currently offered maintenance therapy.

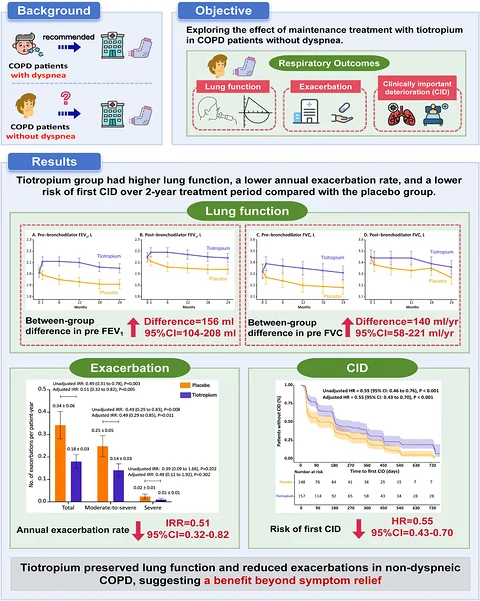

## 1 Introduction

Chronic obstructive pulmonary disease (COPD) is a progressive respiratory disorder characterized by persistent airflow limitation and an enhanced chronic inflammatory response in the airways and lungs [1]. The Global Initiative for Chronic Obstructive Lung Disease (GOLD) guidelines emphasize symptom‐driven management, recommending that all Group A patients (mild symptoms) be offered bronchodilator treatment on the basis of its effect on dyspnea, to be continued if benefit is documented [1]. This approach may limit the long‐term treatment of patients who derive no symptomatic benefit.

A substantial proportion of patients with mild‐to‐moderate COPD, particularly in primary care, do not experience dyspnea [2, 3], and such patients are generally not offered maintenance bronchodilator therapy. Lung function decline and exacerbations nonetheless begin before and independently of dyspnea onset, and the resulting loss of lung function is irreversible [4, 5]. This clinically silent or few‐symptomatic phase poses a treatment problem: Maintenance bronchodilators are not recommended because they cannot relieve symptoms that the patient does not report, yet lung function is already declining. Whether patients without dyspnea benefit from maintenance bronchodilator therapy is, therefore, unknown because previous trials have enrolled symptomatic patients.

Long‐acting muscarinic antagonists (LAMAs), such as tiotropium, are cornerstone therapies in COPD. They demonstrate efficacy in reducing exacerbations, improving quality of life, and preserving lung function in symptomatic populations [6, 7]. Trials, such as UPLIFT, have shown the benefits of tiotropium in moderate‐to‐severe COPD, but data in early‐stage or low‐symptom mild COPD remain limited, with most studies focusing on symptomatic cohorts or broader populations [8, 9]. The tiotropium in early‐stage chronic obstructive pulmonary disease (Tie‐COPD) trial, a multicenter, randomized, and placebo‐controlled study, previously showed that tiotropium maintenance therapy benefits overall patients with early‐stage COPD by attenuating forced expiratory volume in 1 s (FEV_1_) decline and reducing exacerbations [10]. However, gaps persist in understanding its effect on non‐dyspneic subgroups, where symptom relief is irrelevant, and evidence for preventive effects is sparse, highlighting the need for targeted analyses to inform guidelines beyond symptom‐centric approaches [11, 12].

We therefore performed this exploratory post hoc analysis of the Tie‐COPD study to determine whether tiotropium maintenance therapy benefits patients with mild‐to‐moderate COPD who report no dyspnea. We found that, over 2 years of treatment, tiotropium preserved lung function, attenuated the annual exacerbation rate, and delayed the first clinically important deterioration (CID) in this group. These findings are important because they show that measurable benefit can accrue in patients for whom symptom relief is not an achievable treatment goal.

## 2 Results

### 2.1 Baseline Characteristics Were Balanced in the Low‐Symptom Cohort

**FIGURE 1:**
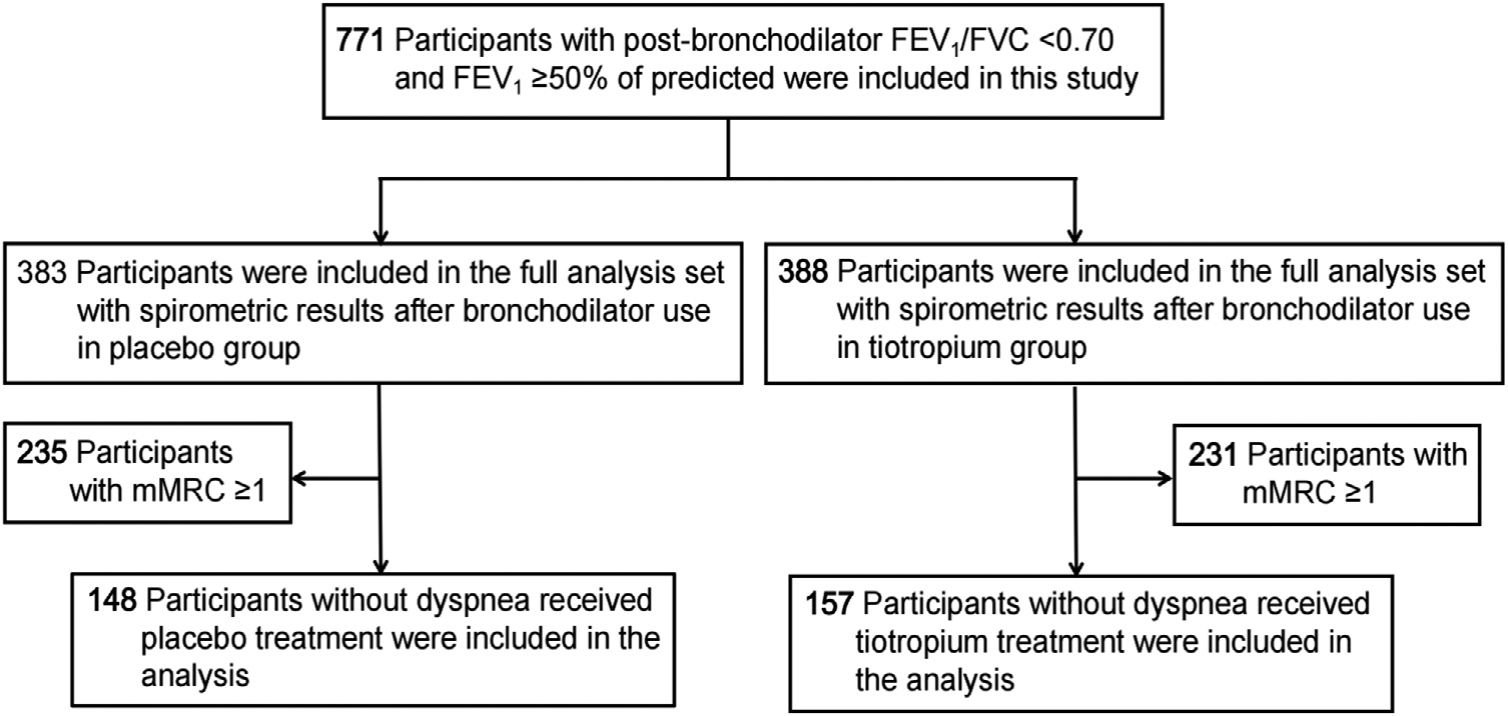
Study flowchart of this study. FEV_1_, forced expiratory volume in 1 s; FVC, forced vital capacity; mMRC, modified Medical Research Council.

Of the 305 non‐dyspneic patients with COPD, 157 received tiotropium and 148 received placebo (Figure 1). The two groups were well‐balanced at baseline (Table 1). The participants were predominantly male (87%), with a mean age of approximately 61 years (standard deviation [SD] 8.4), mild‐to‐moderate airflow limitation, and a low‐symptom burden (mean COPD assessment test [CAT] score 4.2–4.6, mean clinical COPD questionnaire [CCQ] score 0.5–0.6).

**TABLE 1:** Baseline characteristics of participants without dyspnea in the tiotropium in early‐stage chronic obstructive pulmonary disease (Tie‐COPD) study.

Characteristic	Placebo group (*N* = 148)	Tiotropium group (*N* = 157)	*p* value
Age—year	61.4 ± 8.6	61.9 ± 8.2	0.634
Male sex—no. (%)	129 (87)	136 (87)	1.000
Body mass index—kg/m^2^	22.5 ± 2.9	22.6 ± 3.3	0.966
**Smoking status—no. (%)**			0.415
Never smoked	32 (22)	32 (20)	
Former smoking	59 (40)	74 (47)	
Current smoking	57 (39)	51 (32)	
Smoking index—pack‐year	37.40 ± 68.83	34.94 ± 36.21	0.477
Previous respiratory disease other than COPD—no. (%)	6 (4)	10 (6)	0.516
**Pre‐bronchodilator spirometry**			
FEV_1_—liters	2.03 ± 0.54	2.01 ± 0.51	0.871
FEV_1_—% of predicted value	78.66 ± 18.06	78.85 ± 16.32	0.565
FVC—liters	3.33 ± 0.76	3.32 ± 0.72	0.881
FVC—% of predicted value	98.66 ± 18.83	100.45 ± 17.24	0.354
FEV_1_:FVC ratio	60.84 ± 7.11	60.67 ± 7.32	0.974
**Post‐bronchodilator spirometry**			
FEV_1_—liters	2.14 ± 0.52	2.15 ± 0.51	0.890
FEV_1_—% of predicted value	82.90 ± 17.26	84.39 ± 15.74	0.312
FVC—liters	3.45 ± 0.77	3.45 ± 0.70	0.771
FVC—% of predicted value	102.50 ± 19.99	104.14 ± 17.65	0.298
FEV_1_:FVC ratio	62.15 ± 6.56	62.54 ± 6.79	0.384
**GOLD stage—no. (%)**			0.178
Mild (Stage 1)	86 (58)	104 (66)	
Moderate (Stage 2)	62 (42)	53 (34)	
CAT score	4.2 ± 3.9	4.6 ± 4.7	0.763
CCQ score	0.5 ± 0.9	0.6 ± 0.5	0.271

*Note*: Participants without dyspnea are defined as those with a modified Medical Research Council (mMRC) grade of zero. Continuous variables are presented as mean ± standard deviation, and categorical variables are presented as *no*. (%).

Abbreviations: CAT, COPD assessment test; CCQ, clinical COPD questionnaire; COPD, chronic obstructive pulmonary disease; FEV_1_, forced expiratory volume in 1 s; FVC, forced vital capacity; GOLD, Global Initiative for Chronic Obstructive Lung Disease.

### 2.2 Tiotropium Preserved FEV_1_ and Attenuated Lung Function Decline

**FIGURE 2:**
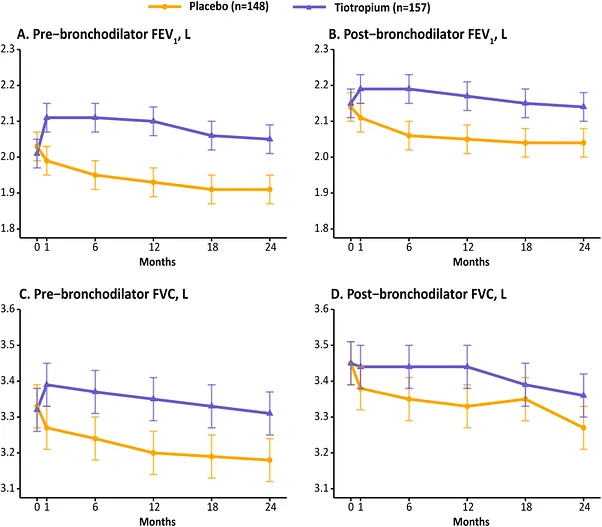
Trend in lung function among the participants without dyspnea in the Tie‐COPD study. Univariable mixed‐effects models were used to estimate the least‐squares means of pre‐bronchodilator FEV_1_ (A), post‐bronchodilator FEV_1_ (B), pre‐bronchodilator FVC (C), and post‐bronchodilator FVC (D) during follow‐up. The models included fixed effects for treatment group, visit, and their interaction, with random intercepts for each participant. FEV_1_, forced expiratory volume in 1 s; FVC, forced vital capacity; Tie‐COPD, tiotropium in early‐stage chronic obstructive pulmonary disease.

**FIGURE 3:**
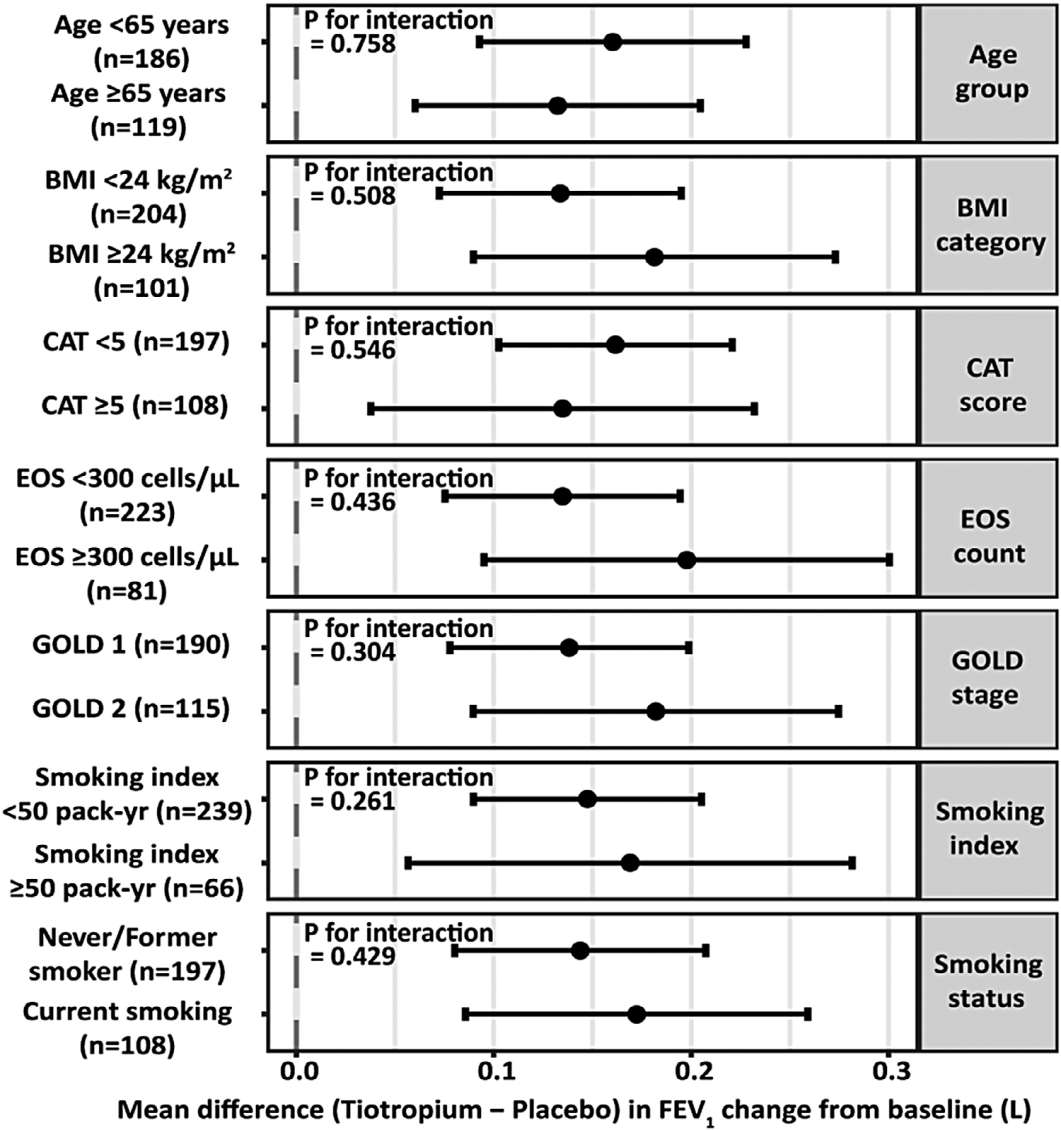
Subgroup analysis of treatment effect on lung function improvement among patients without dyspnea in the Tie‐COPD study. Univariable linear regression models were used to estimate the mean difference (tTiotropium vs. placebo) in pre‐bronchodilator FEV_1_ change from baseline across subgroups. Subgroup variables and cut‐points were age (<65 vs. ≥65 years), BMI (<24 vs. ≥24 kg/m^2^), baseline CAT score (<5 vs. ≥5), blood eosinophil count (EOS; <300 vs. ≥300 cells/µL), GOLD stage (1 vs. 2), smoking index (<50 vs. ≥50 pack‐years), and smoking status (never or former vs. current); the number of participants in each stratum is given in parentheses. *P* for interaction was obtained by adding a treatment‐by‐subgroup interaction term to the corresponding model. These subgroup analyses were exploratory: they were defined post hoc rather than prespecified in a formal statistical analysis plan and were not powered to detect treatment‐by‐subgroup interaction and are therefore presented descriptively. BMI, body mass index; CAT, COPD assessment test; EOS, eosinophil; FEV_1_, forced expiratory volume in 1 s; GOLD, Global Initiative for Chronic Obstructive Lung Disease; Tie‐COPD, tiotropium in early‐stage chronic obstructive pulmonary disease.

Tiotropium maintained higher FEV_1_ and forced vital capacity (FVC) throughout follow‐up compared with placebo (Figure 2, Table S1). Pre‐bronchodilator FEV_1_ differences ranged from 143 to 183 mL (all *p* < 0.001). Post‐bronchodilator FEV_1_ differences ranged from 78 to 112 mL (all *p* < 0.010). In exploratory subgroup analyses by age, body mass index (BMI), CAT score, blood eosinophils, GOLD stage, smoking status, and smoking index, the direction and magnitude of the lung function benefit were similar across subgroups, and no test for treatment‐by‐subgroup interaction approached significance (all *p* for interaction >0.05; range 0.26–0.76; Figure 3). Annual decline rates were significantly attenuated in the tiotropium group (Figure S1). The annual change in pre‐bronchodilator FEV_1_ was −7 ± 8 mL/year with tiotropium versus −55 ± 9 mL/year with placebo (adjusted difference −49 mL/year, 95% confidence interval [CI] −70, −29; *p* < 0.001). Post‐bronchodilator FEV_1_ was −16 ± 8 versus −48 ± 8 mL/year (adjusted difference −34 mL/year, 95% CI −53, −15; *p* < 0.001). Similar patterns were observed for FVC.

### 2.3 Tiotropium Reduced Exacerbations and CID

**FIGURE 4:**
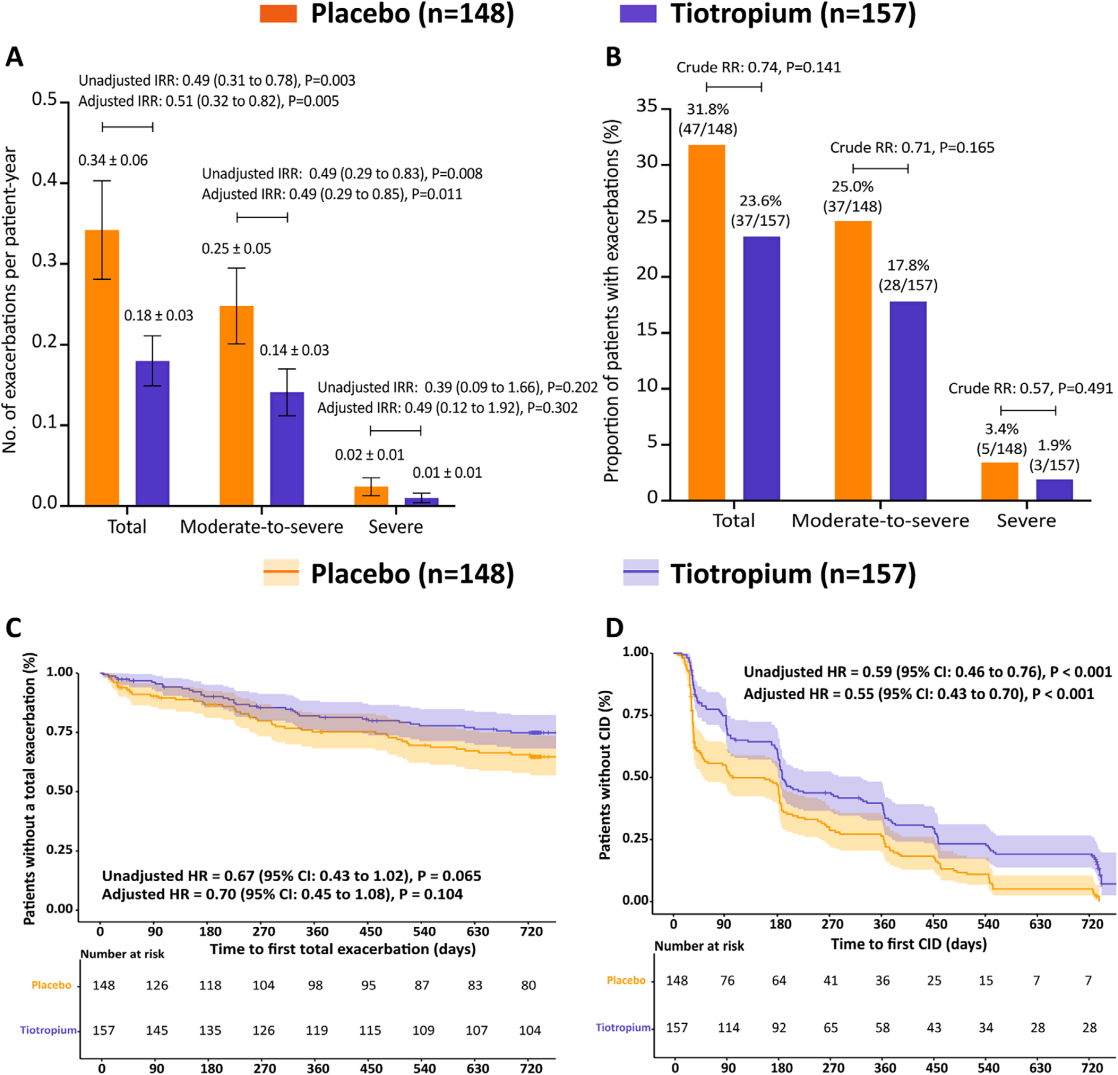
Acute exacerbations and CID among participants without dyspnea in the Tie‐COPD study. Poisson regression models with robust standard errors were used to estimate the incidence rate of exacerbations (A) during follow‐up; bars show the model‐estimated incidence rate and error bars show the standard error of that estimate (tiotropium, *n* = 157; placebo, *n* = 148). Pearson's chi‐square test with continuity correction, or Fisher's exact test when any expected cell count was <5, was used to compare exacerbation proportion between treatment groups (B). Crude RR in the plot (B) is the ratio of exacerbation proportion in the tiotropium group to that in the placebo group. Cox regression models were used to analyze time‐to‐first total exacerbation (C) and time‐to‐first CID (D). All adjusted effects were calculated from models adjusted for age, sex, body mass index, smoking index, and Global Initiative for Chronic Obstructive Lung Disease (GOLD) stage. CI, confidence interval; CID, clinically important deterioration; HR, hazard ratio; IRR, incidence rate ratio; RR, risk ratio; Tie‐COPD, tiotropium in early‐stage chronic obstructive pulmonary disease.

The annual exacerbation rate was lower in the tiotropium group (Figure 4A). The frequency of total exacerbations was 0.18 ± 0.03 per patient‐year versus 0.34 ± 0.06 per patient‐year (adjusted incidence rate ratio [IRR] 0.51, 95% CI 0.32, 0.82; *p* = 0.005); an unadjusted negative binomial model gave essentially the same estimate (IRR 0.49, 95% CI 0.31, 0.79; *p* = 0.003). The frequency of moderate‐to‐severe exacerbations was 0.14 ± 0.03 per patient‐year versus 0.25 ± 0.05 per patient‐year (adjusted IRR 0.49, 95% CI 0.29, 0.85; *p* = 0.011). The proportion of patients with at least one exacerbation tended to be lower in the tiotropium group (Figure 4B). The time‐to‐first exacerbation tended to be longer in the tiotropium group (Figure 4C; unadjusted HR 0.67, 95% CI 0.43, 1.02; *p* = 0.065; adjusted HR 0.70, 95% CI 0.45, 1.08; *p* = 0.104). The time‐to‐first CID was significantly longer (Figure 4D; unadjusted HR 0.59, 95% CI 0.46, 0.76; adjusted HR 0.55, 95% CI 0.43, 0.70; both *p* < 0.001). In the component‐specific analyses (Figure S2), the reduction in CID was driven predominantly by the lung function component (time to first ≥100 mL decrease in pre‐bronchodilator FEV_1_: adjusted HR 0.31, 95% CI 0.23, 0.42; *p* < 0.001), whereas the CAT score deterioration component (adjusted HR 0.92, 95% CI 0.70, 1.21; *p* = 0.55) and the exacerbation component (adjusted HR 0.64, 95% CI 0.39, 1.05; *p* = 0.08) did not individually reach statistical significance.

### 2.4 CAT and CCQ Scores Were Unchanged

No significant between‐group differences were observed in the CAT or CCQ scores over time (CAT score: tiotropium 4.6 vs. placebo 4.8 at Month 24; Table S2), consistent with the non‐dyspneic baseline.

## 3 Discussion

This post hoc analysis of the Tie‐COPD study suggests that, in patients with COPD without dyspnea (modified Medical Research Council [mMRC] Dyspnea Scale score of 0), tiotropium maintenance therapy may preserve lung function, reduce exacerbations, and delay the time‐to‐first CID, despite no change in symptom scores. In this subgroup, tiotropium significantly improved lung function, reduced the exacerbation rate by 49%, and lowered the risk of first CID by 45% (all *p* < 0.01), without any detectable change in CAT or CCQ scores. These findings extend the evidence from the primary Tie‐COPD study, which showed overall benefits in early‐stage COPD, by highlighting the value of early treatment in a strictly non‐dyspneic subgroup [10].

Our findings are consistent with and extend beyond previous long‐term trials, which demonstrated sustained improvement in lung function with tiotropium in patients with moderate‐to‐severe COPD. In UPLIFT, tiotropium provided sustained improvements in pre‐ and post‐bronchodilator FEV_1_ compared with control throughout 4 years of treatment in patients with GOLD Stage 2 (ranging from 87 to 103 mL before bronchodilation and from 47 to 65 mL after bronchodilation) [7, 8]. In our participants without dyspnea, the benefit on lung function was evident as early as 1 month and was sustained throughout the 24‐month treatment period. Our findings extend these observations to patients with mild disease severity and without clinically apparent dyspnea, suggesting that the physiological benefits of tiotropium are not restricted to patients with a substantial symptom burden.

Mechanistically, although this analysis was not designed to test pathways, several are plausible. Sustained muscarinic (M3) blockade produces bronchodilation and reduces hyperinflation and air trapping, directly improving FEV_1_, whereas acetylcholine acting through nonneuronal cholinergic pathways promotes airway inflammation and smooth muscle remodeling, so its blockade may attenuate structural progression [13], and fewer exacerbations may further limit exacerbation‐driven lung function deterioration. The lung function benefit was broadly consistent across baseline strata, including blood eosinophils, GOLD stage, and smoking (Figure 3), and these mechanisms also provide a rationale for greater benefit from early intervention. Nonetheless, they remain hypothetical, requiring dedicated mechanistic and biomarker studies.

The reduction in exacerbations is similarly consistent with prior evidence and is clinically meaningful because even mild exacerbations in mild‐to‐moderate COPD can accelerate lung function decline and increase the healthcare burden [14]. Comparable reductions have been reported in broader COPD populations with tiotropium use [6], but our data emphasize benefits in low‐risk, non‐dyspneic patients who might otherwise forgo maintenance therapy as per the GOLD guidelines [1]. The delayed time‐to‐first CID (HR 0.59) further underscores the role of tiotropium in preventing composite deterioration, consistent with a post hoc analysis in mild‐to‐moderate cohorts where CID risk was reduced by 40%–50% [15], indicating that this preventive effect persists even in patients with minimal symptoms.

The clinical relevance of these physiological findings warrants balanced interpretation. The between‐group difference in pre‐bronchodilator FEV_1_ (143–183 mL) exceeded the ∼100‐mL threshold generally regarded as the minimal clinically important difference (MCID) for FEV_1_ in COPD [16]. However, this comparison should be interpreted cautiously: The ∼100 mL MCID for FEV_1_ is debated and was established mainly in symptomatic patients, and a statistically significant difference in a post hoc subgroup does not by itself prove clinical benefit. These gains in lung function did not improve CAT or CCQ scores, which is expected in a cohort selected for minimal symptoms (mean CAT 4.2–4.6, mean CCQ score 0.5–0.6). A floor effect leaves little room for measurable change, and the between‐group differences stayed well below the CAT MCID of ∼2 [17]. Other factors probably contribute as well: Resting FEV_1_ correlates only weakly with symptom burden in COPD; several CAT and CCQ items, such as energy, sleep, confidence, cough, and sputum, are not expected to respond to bronchodilation; and the analysis was not powered for symptom endpoints in a subgroup of this size. The benefit in this low‐symptom population is thus physiological and preventive—preserving lung function and reducing exacerbations to alter the long‐term trajectory—rather than an immediate symptomatic improvement. Whether this justifies long‐term pharmacotherapy in minimally symptomatic patients cannot be resolved by a post hoc analysis and requires prospective investigation. The component‐specific analysis was consistent with this interpretation; the reduction in CID was driven almost entirely by the lung function component, whereas the CAT score and exacerbation components were individually nonsignificant (Figure S2). Therefore, in this low‐symptom population, the composite primarily reflects lung function preservation and has limited multidimensional sensitivity.

From a clinical standpoint, the results are directly relevant to how maintenance therapy is selected in mild COPD. Current GOLD recommendations adopt a symptom‐driven strategy for initiating and continuing bronchodilator maintenance therapy in mild‐to‐moderate COPD. For Group A patients (low‐symptom burden), bronchodilators are recommended only on the basis of their effect on dyspnea and should be continued solely if symptomatic improvement is documented [1]. In practice, this approach may leave patients without dyspnea (mMRC Dyspnea Scale score 0) outside long‐term pharmacotherapy, because no improvement in absent symptoms can be documented. However, the symptom‐driven strategy rests on the assumption that no symptom improvement equates to no clinical benefit—an assumption with three key limitations in early COPD.

First, the mMRC Dyspnea Scale measures only activity‐related dyspnea on a single ordinal item, and it frequently underestimates the symptom burden compared with multidimensional tools, such as the CAT [18, 19]. Consistent with this, the cohort with an mMRC Dyspnea Scale score of 0 in the present study had a mean CAT score of 4.2–4.6 (Table 1), showing that non‐dyspneic does not mean asymptomatic.

Second, FEV_1_ decline and airway remodeling often begin before patients perceive dyspnea, and the lung function lost during this clinically silent window is largely irreversible. A longitudinal cohort study documented substantial FEV_1_ decline prior to symptom onset [20]. Third, exacerbation risk is largely independent of baseline symptoms. The ECLIPSE study identified a stable “frequent exacerbator” phenotype across all GOLD stages, including patients with minimal symptoms, indicating that exacerbation susceptibility cannot be inferred from current symptom status [21]. Symptom response is therefore an incomplete surrogate for treatment benefit when physiological decline is already underway but not yet apparent to the patient, which provides the rationale for evaluating whether tiotropium confers measurable benefits in patients with an mMRC Dyspnea Scale score of 0.

Extending maintenance therapy to non‐dyspneic patients nonetheless requires weighing cost‐effectiveness, long‐term safety, and patient acceptability. With respect to cost‐effectiveness, COPD exacerbations are major drivers of disease‐related healthcare costs. A prior economic analysis showed that maintenance bronchodilators become progressively more cost‐effective as the proportion of avoided exacerbations increases [22]. Regarding long‐term safety, the 4‐year UPLIFT trial and the subsequent 17,135‐patient TIOSPIR study demonstrated no increase in all‐cause or cardiovascular mortality and a comparable adverse event profile relative to the comparator [7, 23], providing a solid basis for sustained use, even in patients with mild disease. The most challenging element is likely to be patient acceptability and adherence. In the absence of perceived symptoms, motivation for daily inhaled therapy is weaker, and real‐world persistence with tiotropium is known to be influenced by disease severity and comorbidity [24]. This matters because abrupt discontinuation in early COPD leads to rapid loss of the lung function and exacerbation benefits accrued during treatment [25], underscoring the need for structured patient education and shared decision‐making whenever maintenance therapy is initiated in non‐dyspneic patients.

These considerations are especially relevant in primary care, where a substantial proportion of patients with mild COPD are non‐dyspneic [2, 3], yet they are still at risk of progression. Longitudinal data from the European Community Respiratory Health Survey indicate that mild‐to‐moderate COPD with few symptoms progresses to poorer outcomes in smokers, including faster FEV_1_ decline and more hospitalizations [2]. By demonstrating preventive effects in patients without dyspnea, our findings support the broader use of LAMAs, such as tiotropium in early COPD, to alter long‐term trajectories, potentially reducing progression to symptomatic stages [26].

Several limitations of this study should be acknowledged. First, this was an exploratory post hoc subgroup analysis; it was not powered a priori, and it tested multiple endpoints without multiplicity correction, so the findings are hypothesis‐generating. Selection bias was limited because the subgroup was defined by a pre‐randomization characteristic (mMRC Dyspnea Scale score 0), preserving baseline balance (Table 1). Second, the study population was exclusively Chinese and was drawn from a single national trial. COPD in China differs from that in many Western settings in its risk factor profile (including biomass exposure and a high proportion of nonsmokers or light smokers), in body size and spirometry reference values, and in patterns of healthcare access. Ethnic differences in bronchodilator response have also been reported. In addition, patients with COPD in China have a substantially lower BMI than most Western cohorts (mean 22.5–22.6 kg/m^2^ in the present analysis, with 8% of participants below 18.5 kg/m^2^), and a low BMI is a recognized adverse prognostic factor in COPD [1]. A leaner population may, therefore, differ from Western populations both in baseline risk and in the magnitude of benefit obtained from maintenance bronchodilator therapy. Therefore, our findings may not be directly generalizable to other races, regions, or primary care systems, requiring validation in multinational and multiethnic cohorts [27]. Third, exacerbation history in the year before enrollment, which is the strongest predictor of future exacerbations, was not collected in the Tie‐COPD study. Although randomization is expected to balance this variable between the treatment arms so that the relative treatment effect is unlikely to be substantially confounded, its absence precludes accurate baseline risk stratification, may affect the interpretation of absolute exacerbation and CID rates, and prevent risk‐based subgroup analyses. The exacerbation‐related findings should, therefore, be interpreted cautiously. As a sensitivity analysis for unmeasured confounding, the *E*‐values were 3.3 for the exacerbation rate ratio (1.7 at the confidence limit) and 2.8 for CID (2.0 at the confidence limit), so an unmeasured confounder would need associations of this magnitude with both treatment and outcome to explain these results.

Strengths include the randomized, double‐blind, and placebo‐controlled design; the 2‐year follow‐up; and comprehensive multivariable adjustment, which together strengthen the internal validity of the observed treatment effects.

Future studies should validate these outcomes in multinational cohorts and explore biomarkers (e.g., eosinophil levels or imaging) to identify patients without dyspnea who are most likely to benefit. Comparative trials using long‐acting bronchodilator/LAMA combinations in similar subgroups could further inform optimal early therapy [4, 28]. Additionally, assessing the impact of tiotropium on the diffusing capacity of the lungs for carbon monoxide (DLCO) in mild COPD (as low DLCO predicts worse prognosis even in GOLD I) could refine patient selection [29].

In conclusion, this exploratory post hoc analysis showed that tiotropium offers meaningful benefits beyond symptom control in patients with COPD without dyspnea. These hypothesis‐generating findings suggest that a strictly symptom‐based treatment criterion may warrant reassessment, but they require confirmation in an adequately powered prospective trial before they become practice‐changing.

## 4 Materials and Methods

### 4.1 Study Design and Participants

This was a post hoc analysis of data from the Tie‐COPD study, a 2‐year, multicenter, double‐blind, and randomized controlled trial (NCT01455129). Patients with GOLD Stages 1 or 2 COPD were enrolled and randomly assigned to receive tiotropium (18 µg once daily) or matching placebo. The participants completed the CAT, CCQ, and mMRC Dyspnea Scale at baseline, Month 1, and every 3 months thereafter. Spirometry was performed at baseline, Month 1, and every 6 months throughout the treatment period. iFull details of the Tie‐COPD study have been published previously [30].

In the present post hoc analysis, we included participants who did not report dyspnea at baseline, defined by an mMRC Dyspnea Scale score of 0 (Figure 1). Although classified as non‐dyspneic based on an mMRC score of 0, these participants had a measurable, low‐symptom burden (mean baseline CAT score of 4.2–4.6; Table 1) and, therefore, represent a low‐symptom population rather than a truly asymptomatic population. Baseline and 2‐year follow‐up data on demographics, symptom scores, spirometry, and acute exacerbations were analyzed.

The Tie‐COPD trial protocol was approved by the local institutional review board or independent ethics committee of each site, and all participants provided written informed consent before enrollment. This post hoc analysis on the de‐identified data was exempted from ethics review by the institutional review board of The First Affiliated Hospital of Guangzhou Medical University.

### 4.2 Clinical Outcomes

The primary outcome was the between‐group difference in pre‐bronchodilator FEV_1_ over the 2‐year follow‐up period. The secondary outcomes included the annual rate of decline in FEV_1_, the annual incidence rate of acute exacerbations, time‐to‐first CID, and the between‐group differences in CAT and CCQ scores over the follow‐up period.

An acute COPD exacerbation was defined as worsening of at least two major symptoms (cough, sputum volume, sputum purulence, wheezing, or dyspnea) that persisted for at least 48 h, after excluding cardiac insufficiency, pulmonary embolism, pneumothorax, pleural effusion, and cardiac arrhythmia. A moderate event was one that resulted in an outpatient or emergency department visit and regimen modification, including antibiotic agents, oral glucocorticoids, or both. A severe event was one that resulted in hospitalization.

CID was defined as the first occurrence of any of the following events during the treatment period: A decline from baseline in pre‐bronchodilator BD FEV_1_ of ≥100 mL, an increase from baseline of ≥2 U in the CAT score, or a moderate‐to‐severe acute exacerbation.

### 4.3 Statistical Analysis

Continuous variables are presented as the mean ± SD and categorical variables as number (%). Between‐group comparisons at baseline were performed using the independent‐samples *t*‐test or the Mann–Whitney *U* test for continuous variables and the chi‐square or Fisher's exact test for categorical variables. Lung function over time, including the primary outcome (the between‐group difference in pre‐bronchodilator FEV_1_), was analyzed using linear mixed‐effects models, with treatment group, visit, and their interaction as fixed effects and a random intercept for each participant. Least‐squares means at each visit are shown in Figure 2 and Figure S3. Adjusted between‐group differences at each visit (Tables S1 and S2) were obtained from the same models using additional covariate adjustment. The annual rate of lung function decline was estimated using random‐slope linear mixed‐effects models (Figure S1). To assess consistency in the treatment effect, the between‐group difference in the change from baseline in pre‐bronchodilator FEV_1_ was estimated within subgroups defined by age (<65 vs. ≥65 years), BMI (<24 vs. ≥24 kg/m^2^), baseline CAT score (<5 vs. ≥5), blood eosinophil count (<300 vs. ≥300 cells/µL), GOLD stage (1 vs. 2), smoking status (never or former vs. current), and smoking index (<50 vs. ≥50 pack‐years) by linear regression and displayed as forest plots (Figure 3). For each subgroup variable, a treatment‐by‐subgroup interaction term was added to the corresponding model, and the resulting *p* value for interaction is reported in Figure 3. Because this cohort was selected for a low‐symptom burden, the CAT score was dichotomized at 5 rather than at the conventional threshold of 10, at which only 33 participants would have fallen into the higher stratum, and BMI was dichotomized at 24 kg/m^2^ rather than at 18.5 kg/m^2^, below which only 24 participants fell. These subgroup analyses were exploratory and were not prespecified in a formal statistical analysis plan.

Exacerbation rates were compared using Poisson regression, with the logarithm of individual follow‐up time included as an offset so that between‐participant differences in exposure time were accounted for (IRR). Poisson regression was chosen because the outcome was a count of events accrued over person‐time, for which the IRR is the natural effect measure. Because exacerbation counts were over‐dispersed relative to the Poisson assumption (dispersion parameter 2.0 for total exacerbations), robust (sandwich) standard errors were used. An unadjusted negative binomial model was fitted as a sensitivity analysis. The proportion of patients with at least one exacerbation was compared using the chi‐square test (or Fisher's exact test when the expected cell counts were <5) (Figure 4B). The time‐to‐first exacerbation, time‐to‐first CID, and time to each CID component were analyzed by Kaplan–Meier estimation and Cox proportional‐hazards regression (Figure 4C,D; Figure S2). The three components of CID (decrease in pre‐bronchodilator FEV_1_ of ≥100 mL, an increase in CAT score of ≥2, and a moderate‐to‐severe exacerbation) were additionally analyzed separately as time‐to‐first‐event outcomes using the same approach (Figure S2).

Unless otherwise specified, multivariable models were adjusted for age, sex, BMI, smoking index, and GOLD stage, plus the baseline value of the outcome where applicable. Prior exacerbation history was not collected and could not be adjusted for; *E*‐values were calculated to quantify robustness to this and other unmeasured confounding. Mixed‐effects models used all available data under a missing‐at‐random assumption. A two‐sided *p* value of <0.05 was considered statistically significant. All analyses were performed using R version 4.5.1 (R Foundation for Statistical Computing, Vienna, Austria).

## Author Contributions

Full access to all data in the study and take responsibility for the integrity of the data and the accuracy of the data analysis: Zihui Wang and Junfeng Lin. Conception and design: Zihui Wang, Junfeng Lin, Guannan Cai, Fan Wu, Pixin Ran, Yumin Zhou, and Nanshan Zhong. Administrative support: Pixin Ran, Yumin Zhou, and Nanshan Zhong. Provision of study materials or patients: Pixin Ran and Yumin Zhou. Collection and assembly of data: Zihui Wang, Junfeng Lin, Guannan Cai, Fan Wu, Zhishan Deng, Qi Wan, Gaoying Tang, Cuiqiong Dai, Lifei Lu, Kunning Zhou, Xiaohui Wu, Suyin Huang, Fangyan Wu, Nanshan Zhong, Yumin Zhou, and Pixin Ran. Data analysis and interpretation: Junfeng Lin and Zihui Wang. Manuscript writing: Zihui Wang and Junfeng Lin. Critical revision of the manuscript for important intellectual content: Guannan Cai, Fan Wu, Zhishan Deng, Qi Wan, Gaoying Tang, Cuiqiong Dai, Lifei Lu, Kunning Zhou, Xiaohui Wu, Suyin Huang, Fangyan Wu, Nanshan Zhong, Yumin Zhou, and Pixin Ran. All authors have read and approved the final manuscript.

## Funding

This study was supported by the Foundation of Guangzhou National Laboratory (SRPG22‐016 and SRPG22‐018), the National Natural Science Foundation of China (82570065), the Clinical and Epidemiological Research Project of State Key Laboratory of Respiratory Disease (SKLRD‐L‐202402), and the Major Clinical Research Project of Guangzhou Medical University's Scientific Research Capability Improvement Plan (GMUCR2024‐01012).

## Disclosure

The study funding played no role in the study design, data collection, analysis, interpretation of results, writing, or the decision to submit for publication.

## Ethics Statement

The Tie‐COPD trial (ClinicalTrials.gov identifier NCT01455129) was approved by the Ethics Committee of The First Affiliated Hospital of Guangzhou Medical University (approval number 2011‐30) and by the institutional review board or independent ethics committee of each participating site. The present work is a post hoc analysis of deidentified data from that trial; under the policy of our institution (The First Affiliated Hospital of Guangzhou Medical University), post hoc analyses of deidentified clinical trial data are exempt from further ethics review.

## Consent

Written informed consent was obtained from all participants before enrollment.

## Conflicts of Interest

The authors declare no conflicts of interest.

## Supporting information




**Supporting File**: mco271001‐sup‐0001‐SuppMat.docx.

## Data Availability

The dataset collected by this study is not publicly available owing to restrictions in the ethics approval. However, it may be available through communication to the corresponding author and agreement by all coauthors on reasonable request.
