# Supplementary material for: Improvements With Tiotropium Maintenance Therapy in COPD Patients Without Dyspnea
Source: MedComm (2020). 2026 Sep 16;7(10):e71001. doi: 10.1002/mco2.71001 (PMC13583477; doi:10.1002/mco2.71001)
Supplement: Supplementary file 1 [file MCO2-7-e71001-s001.docx]

**Supplementary Information**

**Improvements with Tiotropium Maintenance Therapy in COPD Patients without Dyspnea**

**Running title:** tiotropium in COPD without dyspnea

**Authors**: Zihui Wang^1#^, MD, PhD; Junfeng Lin^1#^, MD; Guannan Cai^1#^, MD; Fan Wu^1,2^, MD, PhD; Zhishan Deng^1^, MD, PhD; Qi Wan^1^, MD; Gaoying Tang^1^, MD; Cuiqiong Dai^1^, MD; Lifei Lu^1^, MD; Kunning Zhou^1^, MD; Xiaohui Wu^1^, MD; Suyin Huang^1,2^, M.D.; Fangyan Wu^1^, MD; Nanshan Zhong^1,2*^, MD, PhD; Yumin Zhou^1,2*^, MD, PhD; Pixin Ran^1,2*^, MD, PhD.

**Institutions and Affiliations:**

1.State Key Laboratory of Respiratory Disease & National Clinical Research Center for Respiratory Disease & National Center for Respiratory Medicine & Guangzhou Institute of Respiratory Health, The First Affiliated Hospital of Guangzhou Medical University, Guangzhou, China.

2.Guangzhou National Laboratory, Guangzhou, China.

\# These authors contributed equally to this work.

* Correspondence to:

Prof. Pixin Ran E-mail: pxran@gzhmu.edu.cn. ORCID: 0000-0001-6651-634X.

Prof. Yumin Zhou E-mail: zhouyumin410@126.com. ORCID: 0000-0002-0555-8391.

Prof. Nanshan Zhong E-mail: nanshan@vip.163.com.

**Contents**

**Table S1. Comparison of lung function between the two treatment groups at different timepoints among the participants without dyspnea in the Tie-COPD Study.**

**Table S2. Difference of CAT score and CCQ score among the participants without dyspnea in the Tie-COPD study.**

**Figure S1. Annual decline in lung function among the participants without dyspnea in the Tie-COPD study.**

**Figure S2. Component-specific analyses of CID among participants without dyspnea in the Tie-COPD study.**

**Figure S3. Trends in symptom scores during follow-up among patients without dyspnea in the Tie-COPD study.**

**Table S1. Comparison of lung function between the two treatment groups at different timepoints among the participants without dyspnea in the Tie-COPD Study.**

| **Follow-up** | **Tiotropium**  **(N=157)** | **Placebo**  **(N=148)** | **Unadjusted** | |  | **Adjusted*** | |
| --- | --- | --- | --- | --- | --- | --- | --- |
|  |  |  | **Difference**  **(Tiotropium – Placebo)** | **P Value** |  | **Difference**  **(Tiotropium – Placebo)** | **P Value** |
| **Pre-bronchodilator FEV_1_** |  |  |  |  |  |  |  |
| Baseline | 2.01±0.04 | 2.03±0.04 | -18 (-139 to 102) | 0.767 |  | -53 (-127 to 21) | 0.162 |
| Month 1 | 2.11±0.04 | 1.99±0.04 | 125 (4 to 246) | **0.043** |  | 143 (93 to 193) | **<0.001** |
| Month 6 | 2.11±0.04 | 1.95±0.04 | 165 (44 to 286) | **0.008** |  | 181 (130 to 233) | **<0.001** |
| Month 12 | 2.10±0.04 | 1.93±0.04 | 167 (46 to 289) | **0.007** |  | 183 (131 to 235) | **<0.001** |
| Month 18 | 2.06±0.04 | 1.91±0.04 | 152 (30 to 274) | **0.015** |  | 168 (115 to 221) | **<0.001** |
| Month 24 | 2.05±0.04 | 1.91±0.04 | 140 (18 to 262) | **0.024** |  | 156 (104 to 208) | **<0.001** |
| **Post-bronchodilator FEV_1_** |  |  |  |  |  |  |  |
| Baseline | 2.15±0.04 | 2.14±0.04 | 8 (-109 to 126) | 0.889 |  | -27 (-97 to 42) | 0.444 |
| Month 1 | 2.19±0.04 | 2.11±0.04 | 83 (-34 to 201) | 0.165 |  | 78 (26 to 129) | **0.003** |
| Month 6 | 2.19±0.04 | 2.06±0.04 | 121 (3 to 239) | **0.045** |  | 112 (60 to 164) | **<0.001** |
| Month 12 | 2.17±0.04 | 2.05±0.04 | 121 (2 to 239) | **0.046** |  | 111 (58 to 164) | **<0.001** |
| Month 18 | 2.15±0.04 | 2.04±0.04 | 116 (-3 to 234) | 0.057 |  | 107 (53 to 160) | **<0.001** |
| Month 24 | 2.14±0.04 | 2.04±0.04 | 105 (-13 to 224) | 0.081 |  | 97 (44 to 150) | **<0.001** |
| **Pre-bronchodilator FVC** |  |  |  |  |  |  |  |
| Baseline | 3.32±0.06 | 3.33±0.06 | -11 (-176 to 153) | 0.895 |  | -38 (-155 to 78) | 0.518 |
| Month 1 | 3.39±0.06 | 3.27±0.06 | 119 (-46 to 284) | 0.157 |  | 127 (49 to 205) | **0.001** |
| Month 6 | 3.37±0.06 | 3.24±0.06 | 135 (-31 to 300) | 0.111 |  | 141 (61 to 221) | **<0.001** |
| Month 12 | 3.35±0.06 | 3.20±0.06 | 155 (-11 to 322) | 0.067 |  | 163 (82 to 243) | **<0.001** |
| Month 18 | 3.33±0.06 | 3.19±0.06 | 136 (-31 to 303) | 0.110 |  | 142 (60 to 224) | **<0.001** |
| Month 24 | 3.31±0.06 | 3.18±0.06 | 134 (-32 to 301) | 0.114 |  | 140 (58 to 221) | **<0.001** |
| **Post-bronchodilator FVC** |  |  |  |  |  |  |  |
| Baseline | 3.45±0.06 | 3.45±0.06 | 3 (-158 to 163) | 0.975 |  | -25 (-141 to 91) | 0.672 |
| Month 1 | 3.44±0.06 | 3.38±0.06 | 55 (-106 to 217) | 0.501 |  | 57 (-22 to 136) | 0.160 |
| Month 6 | 3.44±0.06 | 3.35±0.06 | 91 (-71 to 254) | 0.269 |  | 92 (11 to 173) | **0.025** |
| Month 12 | 3.44±0.06 | 3.33±0.06 | 111 (-52 to 274) | 0.181 |  | 113 (31 to 195) | **0.007** |
| Month 18 | 3.39±0.06 | 3.35±0.06 | 37 (-127 to 200) | 0.657 |  | 38 (-45 to 121) | 0.367 |
| Month 24 | 3.36±0.06 | 3.27±0.06 | 94 (-69 to 257) | 0.257 |  | 95 (13 to 177) | **0.024** |
| **Pre-bronchodilator FEV_1_/FVC** |  |  |  |  |  |  |  |
| Baseline | 60.67±0.67 | 60.84±0.69 | -0.2 (-2.1 to 1.7) | 0.859 |  | -0.7 (-2.2 to 0.8) | 0.341 |
| Month 1 | 62.43±0.68 | 60.47±0.70 | 2.0 (0.1 to 3.9) | **0.044** |  | 2.0 (0.7 to 3.2) | **0.002** |
| Month 6 | 62.72±0.68 | 59.83±0.71 | 2.9 (1.0 to 4.8) | **0.003** |  | 2.8 (1.5 to 4.1) | **<0.001** |
| Month 12 | 62.60±0.69 | 59.91±0.71 | 2.7 (0.7 to 4.6) | **0.007** |  | 2.6 (1.3 to 3.9) | **<0.001** |
| Month 18 | 61.88±0.69 | 59.55±0.72 | 2.3 (0.4 to 4.3) | **0.020** |  | 2.3 (0.9 to 3.6) | **<0.001** |
| Month 24 | 62.18±0.69 | 60.02±0.71 | 2.2 (0.2 to 4.1) | **0.030** |  | 2.1 (0.8 to 3.4) | **0.002** |
| **Post-bronchodilator FEV_1_/FVC** |  |  |  |  |  |  |  |
| Baseline | 62.54±0.67 | 62.15±0.69 | 0.4 (-1.5 to 2.3) | 0.68 |  | -0.1 (-1.5 to 1.2) | 0.855 |
| Month 1 | 64.10±0.67 | 62.27±0.69 | 1.8 (-0.1 to 3.7) | 0.057 |  | 1.4 (0.0 to 2.7) | **0.045** |
| Month 6 | 63.91±0.68 | 61.81±0.70 | 2.1 (0.2 to 4.0) | **0.032** |  | 1.5 (0.1 to 2.9) | **0.030** |
| Month 12 | 64.02±0.68 | 61.41±0.71 | 2.6 (0.7 to 4.5) | **0.008** |  | 2.0 (0.6 to 3.4) | **0.005** |
| Month 18 | 64.04±0.68 | 60.67±0.71 | 3.4 (1.4 to 5.3) | **<0.001** |  | 2.8 (1.4 to 4.2) | **<0.001** |
| Month 24 | 63.32±0.68 | 62.11±0.71 | 1.2 (-0.7 to 3.1) | 0.221 |  | 0.6 (-0.8 to 2.0) | 0.383 |

Plus–minus values are Least Squares Mean ± Standard Error.

* Mixed-effect Models were used to compare lung function between groups at each visit. Models were adjusted for age, sex, body mass index, smoking index, Global Initiative for Chronic Obstructive Lung Disease (GOLD) stage, and baseline lung function value.

**Abbreviation**: FEV_1_, forced expiratory volume in 1 second; FVC, forced vital capacity.

**Table S2. Difference of CAT score and CCQ score among the participants without dyspnea in the Tie-COPD study**

| **Follow-up** | **Tiotropium (N=157)** | **Placebo (N=148)** | **Unadjusted** | |  | **Adjusted*** | |
| --- | --- | --- | --- | --- | --- | --- | --- |
|  |  |  | **Difference**  **(Tiotropium – Placebo)** | **P Value** |  | **Difference**  **(Tiotropium – Placebo)** | **P Value** |
| **CAT score** |  |  |  |  |  |  |  |
| Baseline | 4.61±0.35 | 4.19±0.36 | 0.42 (-0.55 to 1.40) | 0.396 |  | 0.60 (-0.35 to 1.56) | 0.214 |
| Month 1 | 4.07±0.35 | 4.11±0.36 | -0.03 (-1.01 to 0.95) | 0.950 |  | -0.15 (-0.98 to 0.67) | 0.713 |
| Month 3 | 4.14±0.35 | 3.84±0.36 | 0.31 (-0.68 to 1.30) | 0.542 |  | 0.20 (-0.63 to 1.03) | 0.640 |
| Month 6 | 4.65±0.35 | 4.36±0.37 | 0.29 (-0.70 to 1.29) | 0.564 |  | 0.18 (-0.66 to 1.03) | 0.671 |
| Month 9 | 4.27±0.36 | 4.15±0.37 | 0.12 (-0.89 to 1.12) | 0.820 |  | 0.02 (-0.83 to 0.88) | 0.958 |
| Month 12 | 4.61±0.36 | 4.22±0.37 | 0.39 (-0.62 to 1.40) | 0.444 |  | 0.29 (-0.56 to 1.15) | 0.499 |
| Month 15 | 4.26±0.36 | 4.52±0.37 | -0.26 (-1.28 to 0.75) | 0.613 |  | -0.35 (-1.21 to 0.51) | 0.424 |
| Month 18 | 4.50±0.36 | 5.32±0.37 | -0.82 (-1.84 to 0.19) | 0.112 |  | -0.91 (-1.78 to -0.05) | **0.038** |
| Month 21 | 4.76±0.36 | 5.01±0.37 | -0.25 (-1.27 to 0.77) | 0.626 |  | -0.35 (-1.21 to 0.52) | 0.435 |
| Month 24 | 4.61±0.36 | 4.81±0.37 | -0.20 (-1.21 to 0.82) | 0.703 |  | -0.30 (-1.16 to 0.56) | 0.495 |
| **CCQ score** |  |  |  |  |  |  |  |
| Baseline | 0.59±0.04 | 0.54±0.04 | 0.05 (-0.07 to 0.17) | 0.400 |  | 0.07 (-0.04 to 0.18) | 0.214 |
| Month 1 | 0.55±0.04 | 0.61±0.04 | -0.06 (-0.18 to 0.05) | 0.296 |  | -0.08 (-0.18 to 0.02) | 0.127 |
| Month 3 | 0.57±0.04 | 0.60±0.04 | -0.03 (-0.15 to 0.09) | 0.625 |  | -0.04 (-0.15 to 0.06) | 0.399 |
| Month 6 | 0.61±0.04 | 0.61±0.04 | -0.00 (-0.12 to 0.12) | 0.982 |  | -0.02 (-0.12 to 0.09) | 0.767 |
| Month 9 | 0.58±0.04 | 0.65±0.04 | -0.07 (-0.19 to 0.05) | 0.248 |  | -0.08 (-0.19 to 0.02) | 0.125 |
| Month 12 | 0.63±0.04 | 0.65±0.04 | -0.02 (-0.14 to 0.10) | 0.799 |  | -0.03 (-0.14 to 0.08) | 0.594 |
| Month 15 | 0.64±0.04 | 0.69±0.04 | -0.05 (-0.17 to 0.07) | 0.410 |  | -0.06 (-0.17 to 0.04) | 0.244 |
| Month 18 | 0.61±0.04 | 0.74±0.04 | -0.14 (-0.26 to -0.02) | **0.028** |  | -0.15 (-0.26 to -0.04) | **0.007** |
| Month 21 | 0.64±0.04 | 0.72±0.04 | -0.09 (-0.21 to 0.04) | 0.171 |  | -0.10 (-0.21 to 0.01) | 0.074 |
| Month 24 | 0.61±0.04 | 0.68±0.04 | -0.07 (-0.19 to 0.06) | 0.292 |  | -0.08 (-0.19 to 0.03) | 0.143 |
| **mMRC dyspnea score** |  |  |  |  |  |  |  |
| Baseline | 0.00±0.00 | 0.00±0.00 | 0.00 (0.00 to 0.00) | 1.000 |  | 0.00 (0.00 to 0.00) | 1.000 |
| Month 1 | 0.20±0.04 | 0.23±0.04 | -0.04 (-0.16 to 0.08) | 0.524 |  | -0.03 (-0.15 to 0.09) | 0.633 |
| Month 3 | 0.25±0.04 | 0.31±0.04 | -0.06 (-0.17 to 0.06) | 0.358 |  | -0.04 (-0.17 to 0.08) | 0.495 |
| Month 6 | 0.28±0.04 | 0.31±0.04 | -0.03 (-0.15 to 0.09) | 0.613 |  | -0.02 (-0.14 to 0.11) | 0.787 |
| Month 9 | 0.32±0.04 | 0.40±0.05 | -0.08 (-0.20 to 0.04) | 0.200 |  | -0.06 (-0.19 to 0.07) | 0.335 |
| Month 12 | 0.47±0.04 | 0.54±0.05 | -0.07 (-0.19 to 0.05) | 0.268 |  | -0.05 (-0.18 to 0.07) | 0.412 |
| Month 15 | 0.40±0.04 | 0.57±0.05 | -0.18 (-0.30 to -0.05) | **0.005** |  | -0.16 (-0.29 to -0.03) | **0.017** |
| Month 18 | 0.52±0.04 | 0.60±0.05 | -0.09 (-0.21 to 0.04) | 0.166 |  | -0.07 (-0.20 to 0.06) | 0.300 |
| Month 21 | 0.54±0.04 | 0.57±0.05 | -0.03 (-0.15 to 0.10) | 0.656 |  | -0.01 (-0.14 to 0.12) | 0.874 |
| Month 24 | 0.52±0.04 | 0.61±0.05 | -0.09 (-0.22 to 0.03) | 0.136 |  | -0.08 (-0.21 to 0.05) | 0.230 |

Plus–minus values are Least Squares Mean ± Standard Error.

* Mixed-effect Models were used to compare symptom scores between groups at each visit. Models were adjusted for age, sex, body mass index, smoking index, Global Initiative for Chronic Obstructive Lung Disease (GOLD) stage, and baseline symptom score.

**Abbreviation**: CAT, COPD Assessment Test; CCQ, clinical COPD Questionnaire; mMRC, modified Medical Research Council.

**Figure S1. Annual decline in lung function among the participants without dyspnea in the Tie-COPD study**

Linear mixed-effects models were used to estimate the annual decline in pre-bronchodilator FEV_1_ (A), post-bronchodilator FEV_1_ (B), pre-bronchodilator FVC (C), and post-bronchodilator FVC (D) during 2-year follow-up. Adjusted differences and P values were calculated from models adjusted for age, sex, body mass index, smoking index, Global Initiative for Chronic Obstructive Lung Disease (GOLD) stage, and baseline lung function value. Bars show the model-estimated annual rate of change and error bars show the standard error of that estimate; individual participants do not have an observed annual rate of change, so no individual values are plotted (tiotropium, n = 157; placebo, n = 148).

**Abbreviation**: FEV_1_, forced expiratory volume in 1 second; FVC, forced vital capacity; CI, confidence interval.

**Figure S2. Component-specific analyses of CID among participants without dyspnea in the Tie-COPD study.**

Cox regression models were used to analyze time to first clinically important deterioration (A), first FEV_1_ decline of ≥100 mL from baseline (B), first CAT score increase of ≥2 points from baseline (C), and first moderate-to-severe exacerbation (D). Adjusted values were calculated from models adjusted for age, sex, body mass index, smoking index, and Global Initiative for Chronic Obstructive Lung Disease (GOLD) stage.

**Abbreviation:** CID, clinically important deterioration; FEV_1_, forced expiratory volume in 1 second; CAT, COPD assessment test; HR, hazard ratio; CI, confidence interval.

**Figure S3. Trends in symptom scores during follow-up among patients without dyspnea in the Tie-COPD study**

Univariate mixed-effects models were used to estimate the least squares means of CAT score (A), CCQ score (B), and mMRC dyspnea score (C) during follow-up. The models included fixed effects for treatment group, visit, and their interaction, with random intercepts for each participant.

**Abbreviation:** CAT, COPD Assessment Test; CCQ, clinical COPD Questionnaire; mMRC, modified Medical Research Council
